# Frailty impacts immune responses to Moderna COVID-19 mRNA vaccine in older adults

**DOI:** 10.1186/s12979-023-00327-x

**Published:** 2023-01-17

**Authors:** Charles T. Semelka, Michael E. DeWitt, Maria W. Blevins, Beth C. Holbrook, John W. Sanders, Martha A. Alexander-Miller

**Affiliations:** 1 Section on Geriatric Medicine, Department of Internal Medicine, Wake Forest University School of Medicine, Winston Salem, NC USA; 2 Section on Infectious Diseases, Department of Internal Medicine, Wake Forest University School of Medicine, Winston Salem, NC USA; 3 Department of Microbiology and Immunology, Wake Forest University School of Medicine, Winston Salem, NC USA

**Keywords:** Frailty, Immune function, COVID-19

## Abstract

**Background:**

Immune responses to COVID-19 mRNA vaccines have not been well characterized in frail older adults. We postulated that frailty is associated with impaired antibody and cellular mRNA vaccine responses.

**Methods:**

We followed older adults in a retirement facility with longitudinal clinical and serological samples from the first Moderna mRNA-1273 vaccine dose starting in February 2021 through their 3rd (booster) vaccine dose*.* Outcomes were antibody titers, antibody avidity, and AIM+ T cell function and phenotype. Statistical analysis used linear regression with clustered error for antibody titers over multiple timepoints with clinical predictors including, age, sex, prior infection status, and clinical frailty scale (CFS) score. T cell function analysis used linear regression models with clinical predictors and cellular memory phenotype variables.

**Results:**

Participants (*n* = 15) had median age of 90 years and mild, moderate, or severe frailty scores (*n* = 3, 7, or 5 respectively). Over the study time course, anti-spike antibody titers were 10-fold higher in individuals with lower frailty status (*p* = 0.001 and *p* = 0.005, unadjusted and adjusted for prior COVID-19 infection). Following the booster, titers to spike protein improved regardless of COVID-19 infection or degree of frailty (*p* = 0.82 and *p* = 0.29, respectively). Antibody avidity significantly declined over 6 months in all participants following 2 vaccine doses (*p* < 0.001), which was further impaired with higher frailty (*p* = 0.001). Notably, avidity increased to peak levels after the booster (*p* < 0.001). Overall antibody response was inversely correlated with a phenotype of immune-senescent T cells, CD8 + CD28- TEMRA cells (*p* = 0.036, adjusted for COVID-19 infection). Furthermore, there was increased detection of CD8 + CD28- TEMRA cells in individuals with greater frailty (*p* = 0.056, adjusted for COVID-19).

**Conclusions:**

We evaluated the immune responses to the Moderna COVID-19 mRNA vaccine in frail older adults in a retirement community. A higher degree of frailty was associated with diminished antibody quantity and quality. However, a booster vaccine dose at 6 months overcame these effects. Frailty was associated with an increased immune-senescence phenotype that may contribute to the observed changes in the vaccine response. While the strength of our conclusions was limited by a small cohort, these results are important for guiding further investigation of vaccine responses in frail older adults.

**Supplementary Information:**

The online version contains supplementary material available at 10.1186/s12979-023-00327-x.

## Introduction

The COVID-19 pandemic has disproportionately affected the population of nursing home residents, accounting for approximately 25% of the US COVID-19 related deaths, despite making up only 5% of the population of older adults [1–4]. There is a high rate of COVID-19 mRNA vaccination in nursing homes, with over 80% of residents having received at least one booster dose [3, 4]. However, evidence describes waning antibody levels and vaccine effectiveness in older adults compared to young and middle-aged adults [5–9]. Frailty has been correlated with decreased effectiveness of influenza, varicella-zoster, and pneumococcal pneumonia vaccines [10–12]. There is emerging evidence for impaired COVID-19 vaccine responses in community-dwelling frail older adults, but evaluation of immune function was limited to antibody levels without assessment of T cell responses [13–15]. While COVID-19 vaccine immune responses have been studied in the nursing home setting [16–19], the impact of frailty on vaccine responsiveness is not fully understood due to imprecision of frailty measurement [19–26] and limited immunological assessments [15, 27].

Frailty is a geriatric syndrome leading to worsened health outcomes due to impaired regulation of homeostasis, and it serves as a marker of biological aging [28, 29]. This is a common condition with 25-50% of community-dwelling older adults categorized as frail [30, 31]. Unsurprisingly, frailty is a risk factor for nursing home placement [32, 33]. Frailty is also a reliable predictor for adverse health outcomes following COVID-19 infection [34, 35]. Measurement of frailty can be accomplished through clinical assessment of physical health and functional status or through use of a frailty index to quantify accumulation of health deficits [29, 36, 37]. The clinical frailty scale (CFS) is a quantitative frailty measure based upon comprehensive geriatric clinical assessment, and it has been well-validated in COVID-19 research in nursing home populations [35, 36, 38].

Vaccination is an effective public health measure for protection of older adults for whom infectious diseases remain a leading cause of morbidity, mortality, and impaired quality of life [39]. However, changes associated with the aging immune system, termed immune-senescence, lead to impaired vaccine responses. These changes include impaired immune function, e.g., constrained germinal center responses and increased inflammatory subsets of adaptive immune cells [40]. Additionally there are alterations in the immune phenotype of aging T cells, including losses of the proliferative naïve CD8+ cell reservoir replaced by a predominance of terminally differentiated memory CD8+ T cells (TEMRA) and the loss of CD28 and CD27 costimulatory receptors on T cells [41–43]. Immune-senescent changes have been associated with impaired antibody and cellular responses to influenza vaccination in older adults [44–47]. Furthermore, inflammation and impaired tissue repair mechanisms associated with immune-senescent T cells are postulated to contribute to disease pathogenesis, informing a model where immune dysfunction is related to the development of frailty [48–52]. Yet, the interaction of immune-senescence and frailty has not been assessed in association with vaccine responsiveness.

Antibody seroconversion is one of the main measures of vaccine responsiveness. Antibodies are produced by B cells and target specific epitopes on pathogens, which confers protection across variants [19, 22, 53, 54]. Higher quantity antibody titers have been associated with protection from adverse health-related outcomes from COVID-19 and influenza [55–57]. However, frailty and older age have been associated with waning COVID-19 vaccine-elicited antibodies [7, 13, 20, 26, 58].

Importantly, quantitative antibody assays are incomplete descriptors of immunity to SARS-CoV-2 [59]. Qualitative antibody measures, including neutralization and avidity, are critical determinants of protection. Antibody neutralization has been correlated with protection from symptomatic infection, with neutralizing antibodies commonly targeted to the SARS-CoV-2 spike protein receptor binding domain (RBD) [60, 61]. Antibody avidity, a measure of the strength of antibody binding, is an important parameter in effectiveness, including neutralizing capacity of antibody [62, 63]. Avidity assays performed over the vaccine response time course typically describe increases in antibody quality, as B cells with higher-affinity antibodies generated by somatic hypermutation are selected in the germinal center [62, 64].

T cells can provide long-lasting immunity to conserved SARS-CoV-2 epitopes, which facilitates protection from severe disease across variants [65–68]. A practical and highly sensitive approach for measuring T cell responses to SARS-CoV-2 uses activation induced markers (AIM). This assay has been employed to describe the relationship between COVID-19 infection and cellular responses in older adults [69]. Survivors of COVID-19 infection develop improved cellular memory responses following vaccination compared with infection-naïve individuals [26, 70]. However, T cell responses were impaired in older adults compared to younger adults, which is postulated as a contributing mechanism to severe health outcomes associated with COVID-19 disease in the aging population [71–73]. Furthermore, the baseline T cell memory phenotype assessed prior to COVID-19 mRNA vaccination predicts T cell responses to vaccine, with associations of impaired responses in older adults with less naïve T cells [74]. Additionally, impaired influenza vaccine responses are associated with expanded CD8+ CD28- memory T cell populations and reductions in CD4+ T follicular helper cells (T_FH_) in older adults [43–46].

While there is evidence for impaired antibody and cellular COVID-19 vaccine responses with older age, the impact of frailty remains unclear [26, 75]. Here we assessed responses to the Moderna mRNA COVID-19 vaccine by measuring the quantity and quality of vaccine-elicited antibody, as well as, T cell memory phenotype and vaccine-specific responses. In a population of older adults in a retirement community, we tested the hypothesis that the degree of frailty correlates with decreased COVID-19 vaccine immune response.

## Methods

### Study design

Wake Forest University School of Medicine IRB authorized the study and consent forms under IRB#71181. Participants were recruited from a single academic-associated retirement facility. All retirement facility residents were eligible for participation. Exclusion criteria included clinically significant changes in health status including vital sign instability and/or unstable health conditions. Older adults living in the retirement facility, under nursing home care or assisted living, were assessed for the capacity to consent by a physician member of the research team at study enrollment, prior to volunteering written informed consent. Capacity to participate was assessed at each subsequent sample collection.

A cohort of older adults in a retirement facility were followed from the first Moderna mRNA-1273 vaccine dose in February 2021 with blood sample collections at baseline, second dose (4 weeks post first dose), and 2 weeks, 3 months, and 6 months post second vaccine dose*.* A subset of participants was followed for a final blood collection 2 weeks following a third Moderna mRNA-1273 vaccine dose (booster dose). Each study visit blood draw occurred in participant domiciles, which was accompanied by a comprehensive geriatrics clinical assessment with frailty status characterized using the 9-point Clinical Frailty Scale developed by Rockwood [36]. Health conditions, including prior COVID-19 infection, were verified through review of participants’ medical information in the electronic health record (EHR) [EPIC Systems, Madison, WI USA]. The primary outcomes measured antibody titers and avidity to COVID-19 mRNA vaccination. Secondary outcomes measured T cell activation. We conducted exploratory analysis of the association T cell immune phenotype with participant frailty status and age.

### Human sample collection and storage

Sample collections and timeframe were modeled from the Moderna BNT162b2 vaccine study [56]. Plasma was collected using Heparin vacutainer tubes (BD Biosciences). Peripheral blood mononuclear cells (PBMC) were separated from fresh plasma using Ficoll (Cytiva) density gradient centrifugation in Leucosep tubes (Greiner Bio-One) and were cryopreserved in 10% dimethylsulfoxide (DMSO from Sigma) supplemented with fetal bovine serum (FBS from Atlanta Biologicals). Serum was drawn into SST vacutainer tubes containing clotting activator (BD Biosciences) and left at room temperature for 30–60 min, before centrifuging for 10 min at room temperature. Serum and plasma following PBMC separation were aliquoted and frozen at − 80 °C.

### Assessment of humoral responses

#### Elisa

We performed enzyme-linked immunosorbent assays (ELISA) to quantify anti-spike and anti-RBD IgG antibodies from serum and plasma with previously established assays [76]. Antibodies were validated by the manufacturer and titrated for ELISA by serial dilution.

Reagents included phosphate buffered saline (PBS from BioWhittaker, Lonza), Tween 20 (Fisher), TMB for chromogenic development (Sigma-Aldrich), and milk (BD Sciences). Ninety-six half well-plates (Greiner Bio-One) were coated with antigen or PBS for a negative control overnight at 4 °C, then washed with PBS-0.1% Tween and blocked with PBS-3% milk at room temperature for 1 h. Antigens used in ELISA were SARS-CoV-2 Washington-1 spike protein at 12.5 ng/ml or RBD protein at 25 ng/ml (BEI Resources, NIAID, NIH). Aliquoted serum samples were titrated in eight 2-fold serial dilutions in PBS-1% milk starting at a minimum dilution of 1:100, and given high titers in some subjects, starting dilution was increased to a maximum of 1:12,800. Plates were incubated with diluted serum for 2 h at room temperature, washed with PBS-0.1% Tween, and incubated with goat anti-Human IgG HRP (1:4000) (Southern Biotech) detection antibody in PBS-0.1% milk for 1 h at room temperature. Plates were washed with PBS-0.1% Tween and developed with TMB for 30 minutes at room temperature in the dark. The reaction was stopped with 2 N H_2_SO_4_ and the plates were read at 450 nm immediately after stopping. The limit of detection was defined as 1:100 based on the initial dilution tested.

#### Avidity

Quality of antibody binding was assessed with an avidity assay following a previously established procedure [77, 78]. The ELISA assay was performed with spike protein as described above, with modifications as follows. Participant serum was used at a dilution that resulted in half-maximal peak ELISA titer values. Prior to incubation with detection antibody, sodium thiocyanate (NaSCN from Acros Organics) was added at a starting concentration of 5 M with 2-fold dilutions to 0.195 M in PBS per well. PBS alone was added to a negative control well. After a 15 min incubation at ambient temperature, the plate was washed again with PBS-Tween, detection antibody was added, and the ELISA was continued as previously described. The calculation of the avidity index will be described in more detail in the statistical analysis subsection.

### Stimulation and staining of human peripheral blood mononuclear cells

Peptides from Washington-1 SARS-CoV-2 spike protein were obtained from BEI Resources (NR-52402). The entire 181 individual peptides were combined in a peptide mega-pool. Each peptide was resuspended in 70% acetonitrile, pooled, aliquoted, and lyophilized before storing at − 80 C.

PBMC samples were thawed, washed, and resuspended in RPMI media with 5% human albumin and L-Glutamine with between 5 × 10^5^ to 1 × 10^6^ cells per well in 96-well U bottom plates. Cells were cultured for 24 hours in the presence of SARS-CoV-2 spike pooled peptides [0.8 μg/ml of each peptide] or a positive control, 10 μg/mL PHA (Sigma) at 37 °C in a humidified atmosphere containing 5% CO2. Incubation with an equimolar amount of DMSO (Sigma) was performed as negative control.

Surface staining was performed on PBMCs following 24 h stimulation culture. Cells were resuspended in 100 ml PBS with 2% FBS (FACS buffer), then underwent wash and centrifugation between steps, including Live/Dead stain (Biolegend), Fc block (Biolegend), and antibody cocktail stain (antibodies from Biolegend) for 30 minutes at 4 °C in the dark. Following surface staining, cells were washed twice with FACS buffer. After the final wash, cells were resuspended in 200ul PFA fixation buffer.

### Flow Cytometric activation induced cell marker (AIM) assay

We used activation induced surface markers (AIM), a cytokine independent approach, for functional measurement of T cell activation. This technique has previously been reported to be highly sensitive for detection of rare cell populations including circulating T_FH_ cells [79]. We defined a 13-color flow cytometry panel of lymphocyte lineage markers (Supplementary Table 1). Markers of AIM were CD69 + CD137+ for CD8+ T cells and OX40 + CD137+ for CD4+ T cells and T_FH_ cells. T cell memory was described with naïve (CD45RA+ CCR7+), central memory (CD45RA- CCR7+), effector memory (CD45RA- CCR7-), and terminally-differentiated effector memory TEMRA (CD45RA+ CCR7-) populations [43], and the lack of CD28 co-receptor was used as a marker of T cell aging [80].

All samples were acquired on a Fortessa X20 cytometer configured with five excitation lasers (355, 405, 488, 561, 640 nm) and 20 detectable parameters (BD Biosciences, San Diego, CA). Data in .fcs format were exported from the FACSDIVA software of the cytometer and processed directly using FlowJo (version 10.1, FlowJo, LLC).

### Statistical analysis

Data were analyzed using R, version 4.0.3 (R Foundation, Vienna Austria). Figures were created using Prism 9.1.0 (GraphPad Software, San Diego California). A two-sided significance of *P* < 0.05 was considered significant. Sample size was not determined, as this was a convenience sample of a difficult to reach population. We examined the association of log-transformed antibody titers and avidity IC50 values with fixed effects including clinical and demographic variables (age, sex, frailty status, and prior COVID-19 infection) over the vaccine time course. We used linear regression models for estimation of fixed effects with clustered standard errors using the “fixest” R package to account for auto-correlation of an individual’s data over multiple time points. Avidity assays used Prism software to calculate IC50 values for inhibitor response curves to estimate the inhibitor concentration required for half-maximal antibody binding. We used general linear regression models at one timepoint (2 weeks post boost) to analyze the association of clinical and demographic variables with post-booster antibody responses.

Cellular analysis used linear regression models to report results of AIM+ T cells following stimulation with spike pooled peptides with subtraction of DMSO negative control values. We analyzed the relationship of antibody and cellular responses with the geometric mean titer (GMT) of individuals’ antibody titers following 2 vaccine doses in association with AIM+ T cells. Next, we reported the associations of clinical variables with AIM+ T cells in multivariable models. Models for analysis of cellular responses were developed using significant variables from the antibody analysis. Further model selection used stepwise forward selection techniques from the “caret” R package with 10-fold cross validation to minimize overall model root mean squared error (RMSE), used to identify predictors with the highest explanatory power. Additionally, we conducted exploratory analysis on the correlation of participants’ cellular immune-phenotype with demographic features such as, frailty and age, selecting T cell populations associated with older age and impaired vaccine responses from the established immune aging literature [43, 45, 46, 80].

## Results

In a cohort of 15 participants living in retirement facility, median (IQR) age was 90 years (84, 96). Twelve participants were female (80%) and three individuals (20%) had COVID-19 infection prior to vaccination. Participant frailty status was characterized using the CFS frailty scale based on geriatric assessment with a range of mild frailty (*n* = 3), moderate frailty (*n* = 7), and severe frailty (*n* = 5) [Table 1]. Fourteen individuals were followed to 6 months post second vaccine dose. Eight individuals (53%) had blood collected 2 weeks after the third vaccine (booster) dose, and this group had similar distribution of characteristics to the population at baseline [Table 1]. The data were believed to be missing at random, with no observed systematic differences to explain the missing data. The missing antibody and cellular samples occurred due to patient inability/unwillingness to provide samples at a collection time point. Censoring occurred due to death, loss to follow up, and decline in clinical status. One individual developed a SARS-CoV-2 infection (COVID-19) prior to the booster dose and after 6-months post second vaccine dose.

**Table 1:**
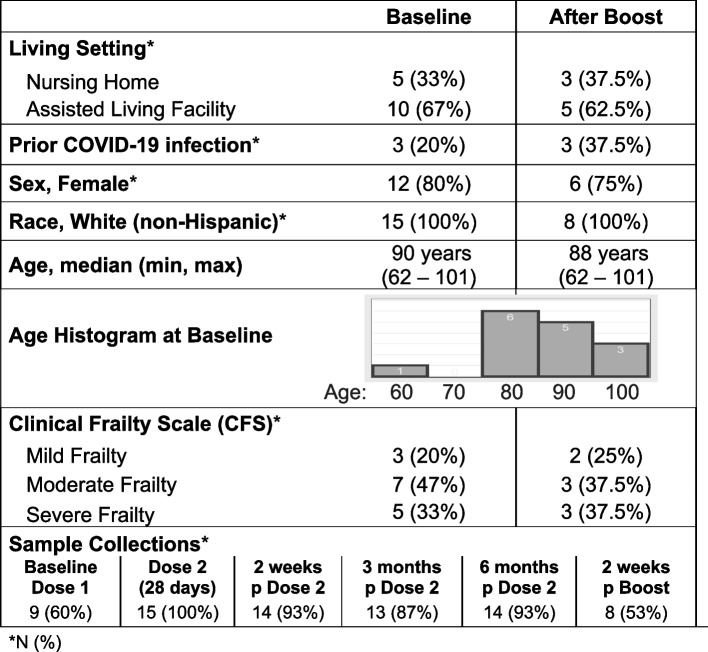
Retirement Community Cohort

### Antibody responses

**Fig. 1:**
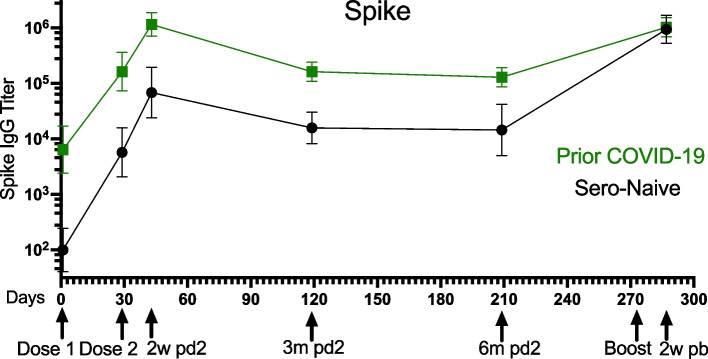
**COVID-19 Impact on Antibody Response in Older Adults.** ELISA was performed on patient samples for antibodies to spike IgG over the vaccine study time course: Dose 1 (baseline), Dose 2 (28 days), 2-weeks post vaccine dose 2 (2w pd2), 3-months (3 m pd2), and 6-months (6 m pd2); Boost, and 2-weeks post boost (2w pb). Over the vaccine time course, Spike IgG antibody titers were 16-fold higher in individuals with prior COVID-19 infection than uninfected (Sero-Naïve) *p* < 0.001. After the booster, titers increased in all individuals but were no longer associated w/ prior infection (*p* = 0.82)

Antibody responses were assessed quantitatively with titers and qualitatively with avidity. The clinical factors of each participant were evaluated in association with their antibody responses over the vaccine time course. Antibody titers over the time course were overall 10-fold higher for spike IgG antibody in individuals with prior COVID-19 infection compared to non-infected individuals (*p* < 0.001) [Fig. 1]. While there were differences in the absolute level of antibody generated, the degree and kinetics of antibody loss measured as percent change from peak (2-weeks) to 6-months post 2nd vaccine dose detected no significant differences by COVID-19 infection status. Following the booster dose, all participants (*n* = 8) had improvement in antibody titers (*p* < 0.001, comparing 6-months post 2nd vaccine dose to 2-weeks post booster vaccine dose). The booster dose overcame the impact of COVID-19 infection on antibody titers, with no significant differences in post-booster antibody levels in individuals with prior COVID-19 compared to uninfected (*p* = 0.82, measured at 2-weeks post booster vaccine dose).

**Fig. 2:**
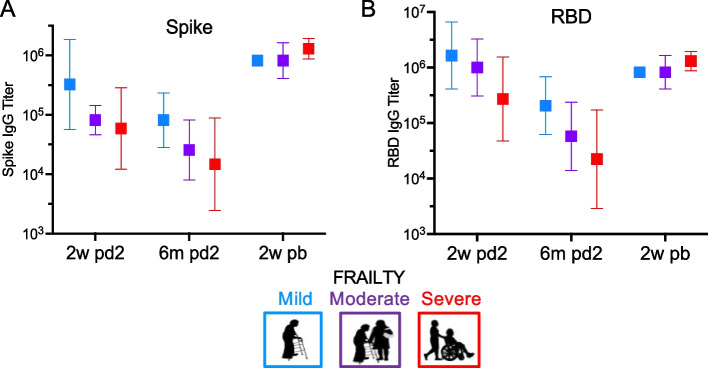
**Frailty Impacts Antibody Response in Older Adults.** ELISA was performed on patient samples for antibodies to spike IgG and RBD IgG over the study time course. Represented data points are: 2-weeks post vaccine dose 2 (2w pd2), 6-months post vaccine dose 2 (6 m pd2), and 2-weeks post booster vaccine (2w pb). (**A**) Individuals with lower frailty compared to higher frailty had 12-fold higher spike protein titers over the time course (*p* = 0.001). After the boost, the impact of frailty status on spike protein titers was no longer significant (*p* = 0.29). (**B**) Individuals with mild frailty compared to severe frailty had 15-fold higher RBD antibody titers over the time course (p < 0.001). After the boost, the impact of frailty status on RBD titers was no longer significant (*p* = 0.66)

The impact of frailty status on antibody titers was evaluated [Fig. 2]. Individuals with milder frailty, compared to severe frailty, had 12-fold higher spike titers (*p* = 0.005) and 15-fold higher RBD antibody titers over the study time course (*p* < 0.001) [Fig. 2 A-B]. When adjusting for prior COVID-19 infection, the effect of frailty on antibody titers remained statistically significant with 12-fold higher spike titers (p = 0.005) and 14-fold higher RBD antibody titers (*p* = 0.002) in less frail individuals. There was an equal distribution of those with prior COVID-19 infection across frailty groups, reducing the risk for biased estimates. We failed to detect a statistically significant association between age or sex with antibody titers in our study population, though our ability to detect differences across these parameters was constrained without a younger control population or equal representation of male sex [data not shown]. The booster vaccine dose overcame the impact of frailty on antibody titers. After the booster dose, there were no statistically significant differences by frailty status on spike (*p* = 0.29) or RBD antibody titers (*p* = 0.79) [Fig. 2 A-B]. After adjusting for prior COVID-19 the impact of frailty remained insignificant on post booster antibody levels for spike (*p* = 0.33) and RBD (*p* = 0.66).

**Fig. 3:**
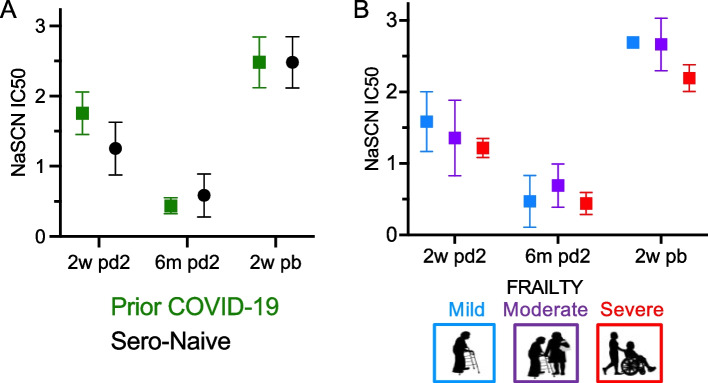
**COVID-19 Vaccine Avidity Response in Frail Older Adults.** ELISA was performed on patient samples for antibodies to spike IgG and RBD IgG over the study time course. Represented data points are: 2-weeks post vaccine dose 2 (2w pd2), 6-months post vaccine dose 2 (6 m pd2), and 2-weeks post booster vaccine (2w pb). (**A**&**B**) After 2 doses of vaccines (from 2w pd2 through 6 m pd2) avidity decreased in all study participants (p < 0.001). After the booster vaccine avidity improved in all participants above vaccine dose 2 levels (p < 0.001). (**A**) Individuals with prior COVID-19 infection had higher overall avidity response than uninfected (Sero-Naïve) (p < 0.001). After the boost, the impact of prior infection was non-significant (*p* = 0.88). Less frail individuals had higher avidity antibody over the study time course (p = 0.001). After the boost, the impact of frailty was no longer significant (*p* = 0.15)

Assessment of antibody quality revealed a significant waning of spike IgG antibody avidity following the second vaccine dose, with a mean 70% reduction in IC50 avidity values measured from 2-weeks to 6-months post 2nd vaccine dose (*p* < 0.001) [Fig. 3]. However, avidity significantly increased after the booster dose, reaching levels 50% higher than the peak observed following the 2nd vaccine dose response (*p* < 0.001). Prior COVID-19 infection was associated with 25% higher avidity antibody over the course of three vaccine doses (p < 0.001). However, after the booster vaccine dose, prior COVID-19 status no longer impacted antibody avidity (*p* = 0.88) [Fig. 3A]. Less frail individuals had approximately 20% higher avidity antibody values compared with severely frail individuals over the study time course (*p* = 0.001 and p < 0.001, unadjusted and adjusted for prior COVID-19, respectively). After the booster, the degree of frailty no longer had significant differences in avidity (*p* = 0.15 and p = 0.15, unadjusted and adjusted for prior COVID-19, respectively) [Fig. 3B].

### Cellular responses

**Fig. 4:**
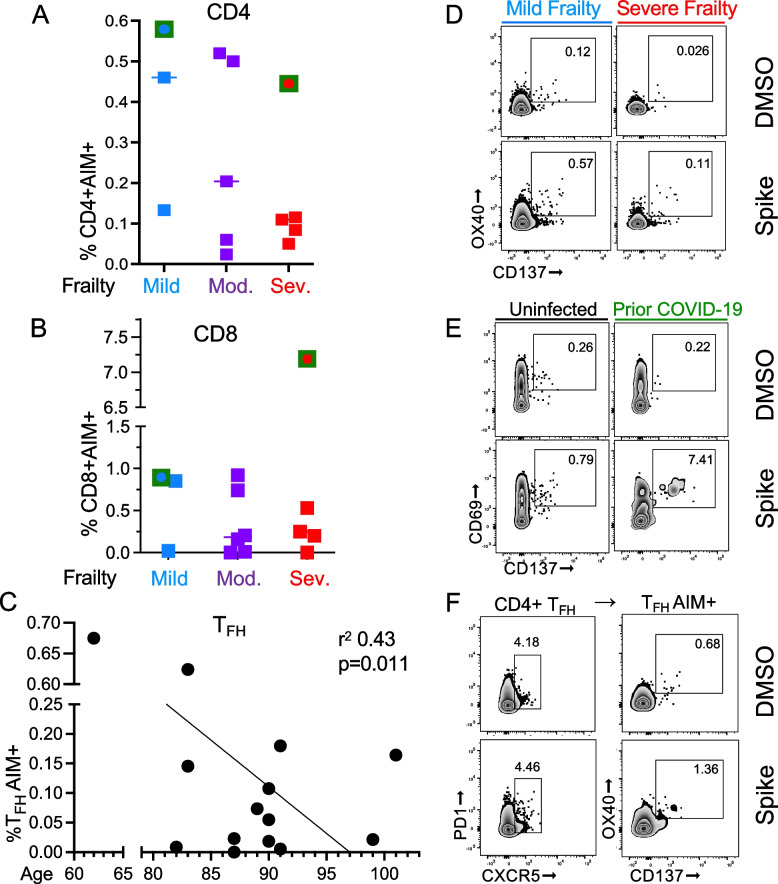
**T Cell Responsiveness in Frail Older Adults**. The flow cytometry AIM assay was used to assess CD4+ and CD8+ T cell responsiveness after stimulation of PBMC from individuals (*n* = 13) collected 2-weeks post vaccine dose 2 for 24 hrs with pooled spike peptides (0.8μg/mL) or equimolar DMSO negative control. (**A**) While individuals with prior COVID-19, indicated by a green square (*p* = 0.055) had a trend towards increased AIM+CD4+ cells, frailty was not a significant predictor (*p* = 0.13). (**B**) Prior COVID-19 infection (green square) was positively associated with AIM+CD8+ cells (*p* = 0.0045), but frailty was not a significant predictor (*p* = 0.17). (**C**) Older aged individuals had decreased AIM+ T_FH_ cells (*p* = 0.011). Representative flow plots with spike and DMSO negative control of (**D**) CD4+ AIM by mild (blue line) or severe (red line) frailty status, (**E**) CD8+ AIM by uninfected (black line) or prior COVID-19 (green line), and (**F**) CD4+ T_FH_ cells, defined by CD4+ CD4 + CXCR5 + PD1+ cells, and AIM defined by OX40 + CD137 +

We analyzed spike-specific cellular responses in *n* = 13 (87%) of participants at peak response to the 2nd vaccine dose (2-weeks post). Of the participants analyzed in this group, 2 individuals had prior COVID-19. For this study, PBMC were stimulated with peptide pools from the SARS-CoV-2 spike protein. Responding cells were determined by increases in CD69 + CD137+ (CD8+ T cells) or OX40 + CD137+ (CD4+ T cells) [representative data are shown in Fig. 4D-E, and gate selection is shown in Supplementary Fig. 1]. Clinical variables with significant impact on antibody responses were selected to predict vaccine-specific T cell responses. Individuals with prior COVID-19 infection had mean detection of 4.5% AIM+CD8+ cells compared with 0.3% in uninfected individuals (SD 1.1, *p* = 0.005). In those with prior COVID-19 infection, AIM+CD4+ cells were detected 0.6% compared with 0.3% in uninfected individuals (SD 0.14, *p* = 0.072). Frailty was not independently associated with AIM+CD8+ or AIM+CD4+ cell responses (*p* = 0.45 and *p* = 0.13, respectively). When controlling for prior COVID-19, the association of frailty with vaccine-specific AIM+CD8+ and AIM+CD4+ T cells remained non-significant (*p* = 0.17 and *p* = 0.12, respectively) [Fig. 4A & B]. Circulating T follicular cells (T_FH_) cells, a subset of CD4+ T cells defined by CD4 + CXCR5 + PD1+ with OX40 + CD137+ for AIM, were selected for analysis because T_FH_ cells assist in antibody production via B cell maturation. AIM+ T_FH_ cell responses were not associated with prior COVID-19 or frailty (*p* = 0.50 and *p* = 0.81, respectively) [data not shown]. However, with machine learning model selection to identify predictors, we found an association between age and AIM+ T_FH_ cell responses, with a 0.15% increase in T_FH_ response for every 10 years of age younger in participants (SD 0.05, *p* = 0.011) [Fig. 4C]. We did not see a relationship between age and AIM+CD8+ or AIM+CD4+ T cells in our study population.

We examined the relationship between vaccine-specific T cell responses and antibody responses. The total antibody response to 2 vaccine doses was calculated using the geometric mean of titers from baseline through 6-months post 2nd vaccine dose. Increases in vaccine-specific AIM+CD8+ and AIM+CD4+ cells, were positively associated with the total antibody response (*p* = 0.022, r^2^ = 0.39 and *p* = 0.019, r^2^ = 0.41; respectively) [data not shown]. AIM+ T_FH_ cell responses were not associated with the total antibody response, calculated as above (*p* = 0.4) [data not shown].

**Fig. 5:**
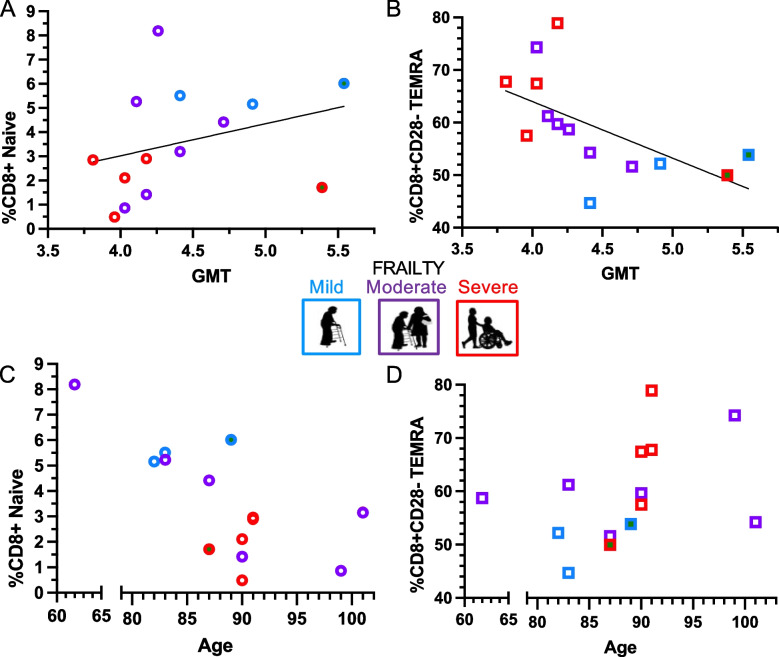
**T Cell Memory Phenotype Association with Antibody Response and Frailty.** T cell memory populations were associated with total antibody response (GMT) and clinical factors of frailty status and age. T cells were collected 2-weeks post vaccine dose 2, and CD8+ naive cells (CD45RA+ CCR7+) and CD8 + CD28- TEMRA cells (CD45RA+ CCR7- CD28-) evaluated based on associations with aging and impaired vaccine response in the literature. (**A**&**B**) The total antibody response to 2 vaccine doses, was calculated with the geometric mean of antibody titers (GMT) to 2 vaccine doses (from baseline to 6-months post vaccine dose 2). (**A)** CD8+ naive cells had a positive trend with total antibody response (*p* = 0.092, r^2^ = 0.77; adjusted for prior COVID-19, as indicated by a central green dot) (**B**) There was a negative association of CD8 + CD28- TEMRA cells with total antibody response (*p* = 0.028, r^2^ = 0.81; adjusted for COVID-19). (**C**&**D**) Adjusting for prior COVID-19 (indicated by a central green dot), (**C**) CD8+ naïve cells were decreased with higher frailty (*p* = 0.026) and older age (p = 0.004), and (**B**) CD8+ CD28- TEMRA cells were increased with severe frailty (*p* = 0.056), but not older age (*p* = 0.31)

Additionally, we characterized the T cell memory phenotype in our study cohort to predict vaccine antibody responses. Based on reported alterations in T cell memory phenotype associated with aging and impaired antibody responses to the influenza vaccine [43, 45, 46], CD8+ naive cells (CD45RA+ CCR7+) and CD8 + CD28- TEMRA cells (CD45RA+ CCR7- CD28-) were selected for assessment. The T cells used in the memory phenotype analyses were collected at 2-weeks post 2nd vaccine dose. The total antibody response to 2 vaccine doses, calculated as above, had a positive trend with CD8+ naive cells (*p* = 0.28, r^2^ = 0.09 and *p* = 0.092, r^2^ = 0.77; unadjusted and adjusted for prior COVID-19 respectively) [Fig. 5A]. There was a negative association of CD8 + CD28- TEMRA cells with total antibody response to 2 vaccine doses (*p* = 0.027, r^2^ = 0.35 and *p* = 0.028, r^2^ = 0.81; unadjusted and adjusted for COVID-19 respectively) [Fig. 5B].

Furthermore, we investigated the relationship of the clinical factors of frailty and age with participants’ T cell memory phenotype. Individuals with more severe frailty had a smaller population of naïve CD8+ cells, with the mean percent of naïve cells in total CD8+ population, of 5.6% in those with mild frailty and 2.5% in those with severe frailty (SD 0.7, *p* = 0.019) [Fig. 5C]. Older age was also associated with less CD8+ naïve cells (1.8% decrease for each 10 years of age older in participants (SD 0.5, *p* = 0.003) [Fig. 5C]. After adjusting for prior COVID-19, the impact of frailty and age on CD8+ naïve cells remained significant (*p* = 0.026 and *p* = 0.004, respectively). Individuals with more severe frailty had a higher proportion of CD8 + CD28- TEMRA cells, with a mean percent of total CD8+ population, of 50% in those with mild frailty in comparison to 73% in those with severe frailty (SD 3.1, *p* = 0.048), but older age lacked the same association (*p* = 0.32) [Fig. 5D]. After adjusting for prior COVID-19, the estimated impact of frailty and age remained similar on CD8 + CD28- TEMRA cells (*p* = 0.056 and *p* = 0.31, respectively).

We conducted exploratory analysis across all the T cell memory populations for correlation of immune-senescence markers with clinical factors, including frailty, age, and functional decline. In validation of the preceding analysis, participant frailty status had the strongest negative correlation with CD8+ naive cells (r = − 0.61) and positive correlation with CD8 + CD28- TEMRA cells (r = 0.54) [Supplemental Fig. 2]. Older age had strong negative correlations with CD8+ naive cells (r = − 0.74) and CD4+ T_FH_ cells (r = − 0.59) [Supplemental Fig. 2]. Interestingly, individuals who experienced functional decline and increased frailty during the study period (*n* = 7) were found to have positive correlations with CD4 + CD28- TEMRA cells (r = 0.68) regardless of their baseline frailty status [Supplemental Fig. 2].

**Fig. 6:**
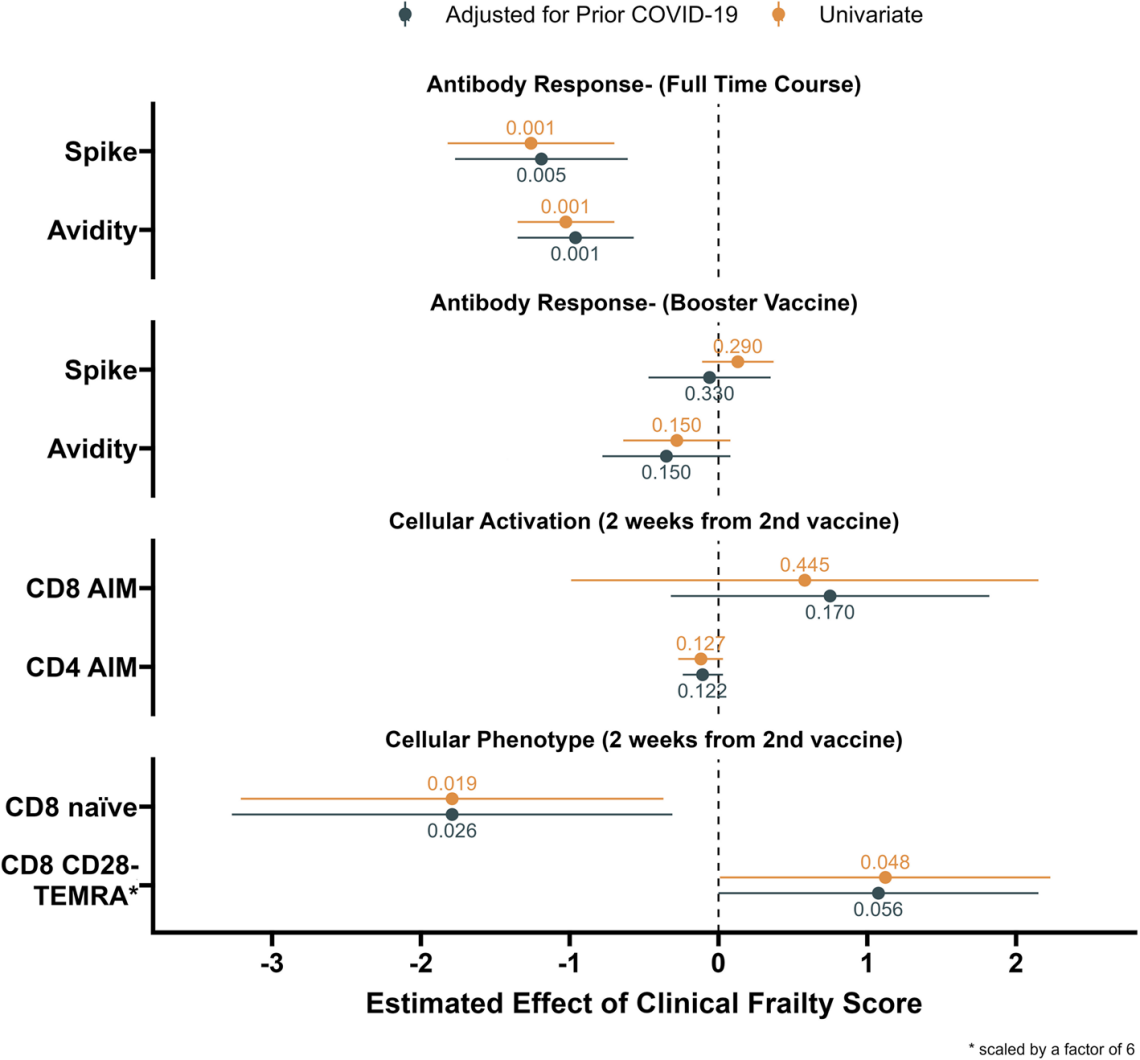
**Frailty Effects on Immune Responses to COVID-19.** The estimated effect sizes and significance values for the impact of CFS frailty score on the assay results of this study. Estimates were provided for the unadjusted and adjusted effect of frailty, controlling for the effect of COVID-19 infection. Central points represent estimated effect size with whiskers to represent 95% CI. A *p*-value is provided with each estimate. Spike protein antibody and avidity over the full time course was impaired in individuals with higher frailty. The booster vaccine dose overcame the effects of frailty on spike antibody titers and avidity. CD4 + AIM and CD8 + AIM vaccine-specific responses were not significantly impacted by frailty. There was an association of higher frailty with a T cell memory phenotype of decreased CD8+ naïve and increased CD8 + CD28- TEMRA cells. The estimated effect of frailty on CD8 + CD28- TEMRA cells was scaled by a factor of 6 to fit on the figure

Finally, we provided a comprehensive illustration of the relationship between frailty and COVID-19 vaccine antibody and cellular responses found in our study population. We created a forest plot with estimated effect sizes and significance values for the association of frailty with the assays we described above [Fig. 6]. We found a pattern of impaired antibody response with increased frailty, which improved with a booster vaccine dose. Vaccine-specific CD4 + AIM+ and CD8 AIM+ T cell responses were not impacted by frailty. However, there was a relationship between frailty with the T cell memory phenotype of decreased CD8 naïve and increased CD8 CD28- TEMRA cells. Changes in T cell memory phenotype were associated with impaired overall antibody response in our analyses.

## Discussion

In this small retirement community cohort, we characterized the adaptive immune responses of frail older adults to the initial series of the Moderna COVID-19 mRNA vaccine. Overall, the study participants generated high levels of antibody following vaccination that reached similar levels following a booster vaccine, regardless of frailty status. As expected, in individuals with prior COVID-19 infection antibody quantity was higher at baseline and reached a higher peak after the 2nd vaccine dose. However, the advantage in spike IgG antibody level afforded by prior infection was lost following the booster dose.

A higher level of frailty was associated with decreased antibody quantity over the vaccine series time course. The full impact of age was unable to be verified as our study population lacked younger adult controls, but within the very old population we found frailty was a better predictor than age for antibody quantity. While our small sample size limited our ability to refine this relationship, these findings are in agreement with studies of the Zoster vaccine [11]. Importantly, the booster vaccination appeared to overcome the effects of frailty on spike and RBD protein antibody quantity. Studies of frail community-dwelling adults have similarly reported COVID-19 booster vaccine doses overcoming impaired antibody responses [15]. This suggests maximal generation of antibody can be reached with appropriate boosting even in frail older adults.

Surprisingly, antibody avidity significantly waned over the 6 months following the second vaccine dose for all older adult participants. These results contrast with findings in younger healthy adults where anti-spike IgG avidity increased over 6 months following the second vaccine dose, which was accompanied by persistent germinal center (GC) reactions in responding B cells [64]. The decrease in avidity observed in our study is consistent with attenuated GC reactions in older individuals resulting in lower quality antibody secreting cells following vaccination similar to findings of impaired GC responses and vaccine-specific antibody generation in older adults following influenza vaccination [40, 81]. Remarkably, anti-spike IgG avidity increased rapidly after the boost. The increase in avidity early after boosting suggests either activation of higher avidity memory B cells that originated from the initial vaccine doses or rapid affinity maturation following the boost. As with antibody quantity, increased frailty may be tied to impaired antibody avidity. While these results are intriguing, the small number of individuals used here requires additional study to fully define vaccine-elicited B cell responses and antibody production in older adults represents an important area for future study.

To gain insights into cellular responsiveness to COVID-19 vaccination, we analyzed the spike-specific activation of T cells after 2 vaccine doses in our study population. We found an increase in spike-specific T cell responsiveness in individuals with prior COVID-19 infection. We saw no evidence of frailty status impact on vaccine elicited CD4+ or CD8+ T cell responses. Interestingly, older age, but not frailty status, had a negative association with T_FH_ cell spike-specific activation. Spike-specific activation of CD4+ and CD8+ T cells was associated with overall antibody responses.

Based on the literature of immune-aging, we analyzed key features of immune-senescence in our study population, including a depletion of CD8+ naïve cells, which has been associated with aging [43, 46], and increased CD8 + CD28- TEMRA cells, which has been associated with impaired influenza vaccine response in older adults [45, 46]. We found an increased proportion of CD8 + CD28- TEMRA cells was independently associated with impaired COVID-19 vaccine antibody response. After adjusting for the effects of a prior COVID-19 infection, individuals with more CD8+ naïve cells trended towards improved antibody response. The certainty of these results is limited by the small study sample size; however, our findings are consistent with evidence suggesting a greater baseline population of naïve T cells predicts improved COVID-19 vaccine responses [74]. There is biological plausibility to this argument as immune-senescent changes, particularly within the repertoire of TEMRA T cells, can promote unregulated inflammatory immune responses [43, 50].

Additionally, we explored the associations of clinical factors including frailty and age with cellular immune-senescence. Individuals with more severe frailty had fewer naïve CD8+ cells and increased CD8 + CD28- TEMRA cells. While older aged individuals had fewer naïve CD8+ cells, the relationship between age and CD8 + CD28- TEMRA cells was not significant. However, older age was uniquely associated with a decreased T_FH_ cell population, suggesting distinct contributions of frailty and age on the aging immune system. While these findings need further exploration due to a small sample size, they suggest for individuals in the later years of life, frailty may be more predictive than age for immune responsiveness to vaccine.

### Strengths and weaknesses

We addressed a range of questions in the field of aging immunology using a study design including sample collections, assay selection, and timeframe modeled after the phase 3 trial results published from the Moderna vaccine group [50, 56]. We investigated the antibody and T-cell response to a novel vaccine, where the window is closing for further analysis of immune-dynamics in an unexposed population to SARS-CoV-2 antigen. While chronological age of older adults is often examined in vaccine immunology publications, there has been little focus on the interplay between chronological age and frailty.

Reliable measures of frailty including the clinical frailty scale (CFS) have been described in a restricted number of nursing home COVID-19 vaccine studies [26, 27, 58]. The results of such studies have been diminished by minimal variability in frailty status, which constrains the ability to detect the association of frailty with vaccine responses [26], short follow up time [27], and limited immunological assays, without assays for quality of antibody or cellular vaccine responses [57]. As waning response to COVID-19 vaccination has been described, it is of particular importance to guide booster vaccine recommendations for frail older individuals [82]. While there is debate regarding the role of immune-senescence in impaired vaccine responses across the population of older adults, our study describes frailty, as a marker of biological aging, is likely an important factor when considering immune-senescence with antibody and cellular immune responses to COVID-19 mRNA vaccine [74, 83].

The main limitations of our study are the small sample size, including loss of study participants at the booster vaccine sample collection. With this small sample size, it is difficult to firmly establish the effects of age and frailty, though the consistent association of frailty with impaired COVID-19 vaccine responses across our assays, with supporting evidence in the literature, lends credibility to our findings. We lacked a younger, healthy control population, which limited our assessment of the full impact of age and frailty on vaccine response. Our population’s assessments on the Clinical Frailty Scale, from mildly frail to severely frail, did not capture the full range of clinical and functional health status. Furthermore, our cohort was recruited from a retirement community, which constrains the generalizability of our findings to this setting. These aspects of our study design introduce limitations on the statistical power and significance of our findings. We used linear regression models with clustered error to measure individual participant antibody responses over time, and though a valid statistical method, a few individuals with extreme responses may have inflated the estimates of our results. However, the equal distribution of prior COVID-19 infection and relatively equal representation across levels of frailty reduced the chance for error. The assays were selected for their rigorous performance, but we did not include cytokine release assays to bolster cellular analysis or analysis of responses to SARS-CoV-2 variants, including the Omicron lineage. We did not study cellular response after the booster dose, but T cell activation has been reported to remain stable between the second and the first booster COVID-19 mRNA dose in older individuals [70, 84].

## Conclusion

Our findings reflect new insights into the immune response to COVID-19 vaccination in frail older adults. Frailty was associated with impaired vaccine responses across the adaptive immune system, spanning decreased antibody titers and avidity, as well as an increased immune-senescent phenotype of memory T cells. Importantly, the booster dose improved antibody titers in all older adults, overcoming the effects of frailty or prior COVID-19 infection. We found waning of antibody avidity at 6 months following the second vaccine dose across our study population, particularly in the most frail. We speculate this may be the result of altered germinal center function compared to younger healthy adults [64]. However, a booster vaccine dose promoted increases in antibody avidity regardless of frailty or prior COVID-19 infection, which surpassed the magnitude of the avidity response from the second vaccine dose.

We found impaired COVID-19 vaccine antibody response was associated with increased terminally differentiated CD8+ T cells, as has been described in the influenza vaccine literature [45, 46]. Terminally differentiated CD8+ T cells were also associated with a higher degree of frailty in participants. These data support a model where the immune-senescence and frailty are interrelated, resulting in impaired immune responses to vaccine [50, 75]. While the conclusions from our small cohort study warrant further investigation, our results are in line with the current evidence [15, 74]. Together our data show for individuals in the later years of life, frailty is independently related to impaired COVID-19 vaccine responses and alterations in the aging immune system. Understanding the aging immune system opens the door to important guidance for a healthy aging population, including booster vaccine recommendations and interventions to reduce frailty [85].

## Supplementary Information

**Additional file 1.** Supplementary Fig. 1. Sample Gating Strategy. Supplementary Fig. 2 A heat map was used to represent correlation of clinical factors (frailty, age, and functional decline) with T cell memory populations: naïve, central memory (CM), effector, and terminally-differentiated effector memory (TEMRA), and CD28- was used as a marker of T cell aging. The strength of relationship was represented pictographically with boxes (blue is positive, red is negative) and numerically with correlation coefficients. Key results are starred in white, including frailty had the strongest positive correlation with CD8+ TEMRA CD28- cells (r = 0.54) and negative correlation with CD8+ naive cells (r = − 0.61). Age was negatively correlated with CD8+ naive cells (r = − 0.74) and CD4+ T_FH_ cells (r = − 0.59). Individuals with increased frailty over the study period, regardless of baseline characteristics, had strong correlations with CD4+ TEMRA CD28- cells (r = 0.68). Supplementary Table 1
