# Supplementary material for: Frailty impacts immune responses to Moderna COVID-19 mRNA vaccine in older adults
Source: Immun Ageing. 2023 Jan 17;20:4. doi: 10.1186/s12979-023-00327-x (PMC9843107; doi:10.1186/s12979-023-00327-x)
Supplement: Supplementary file 1 — Additional file 1. Supplementary Fig. 1. Sample Gating Strategy. Supplementary Fig. 2 A heat map was used to represent correlation of clinical factors (frailty, age, and functional decline) with T cell memory populations: naïve, central memory (CM), effector, and terminally-differentiated effector memory (TEMRA), and CD28- was used as a marker of T cell aging. The strength of relationship was represented pictographically with boxes (blue is positive, red is negative) and numerically with correlation coefficients. Key results are starred in white, including frailty had the strongest positive correlation with CD8+ TEMRA CD28- cells (r = 0.54) and negative correlation with CD8+ naive cells (r = − 0.61). Age was negatively correlated with CD8+ naive cells (r = − 0.74) and CD4+ TFH cells (r = − 0.59). Individuals with increased frailty over the study period, regardless of baseline characteristics, had strong correlations with CD4+ TEMRA CD28- cells (r = 0.68). Supplementary Table 1 [file 12979_2023_327_MOESM1_ESM.pdf]

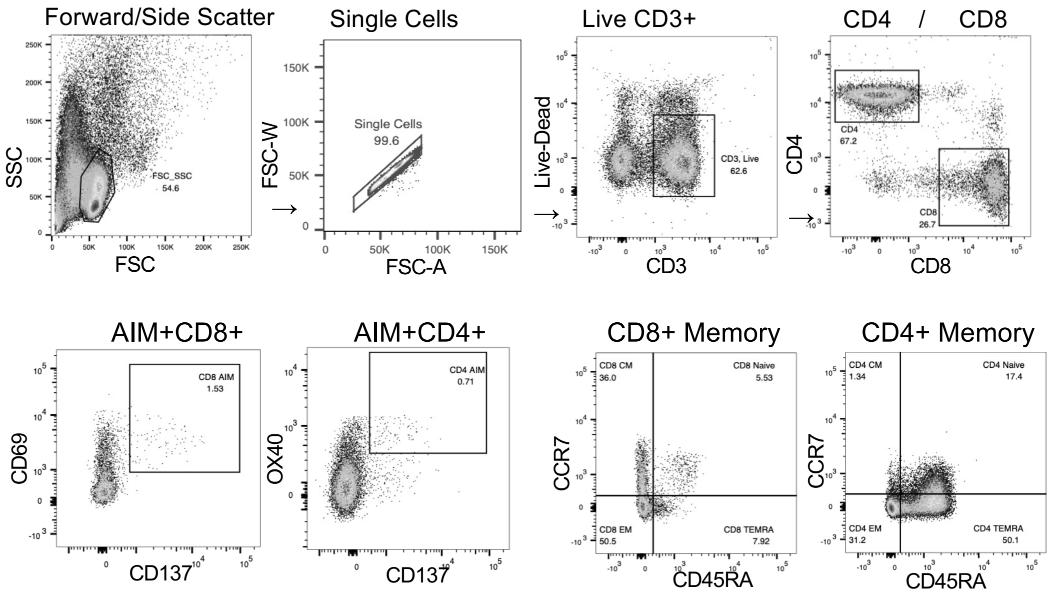
Forward/Side Scatter
SSC
FSC
FSC_SSC
54.6
Single Cells
FSC-W
FSC-A
Single Cells
99.6
Live CD3+
Live-Dead
CD3
CD3, Live
62.6
CD4 / CD8
CD4
CD8
CD4
67.2
CD8
26.7
AIM+CD8+
CD69
CD137
CD8 AIM
1.53
AIM+CD4+
OX40
CD137
CD4 AIM
0.71
CD8+ Memory
CCR7
CD45RA
CD8 CM
36.0
CD8 Naive
5.53
CD8 EM
50.5
CD8 TEMRA
7.92
CD4+ Memory
CCR7
CD45RA
CD4 CM
1.34
CD4 Naive
17.4
CD4 EM
31.2
CD4 TEMRA
50.1

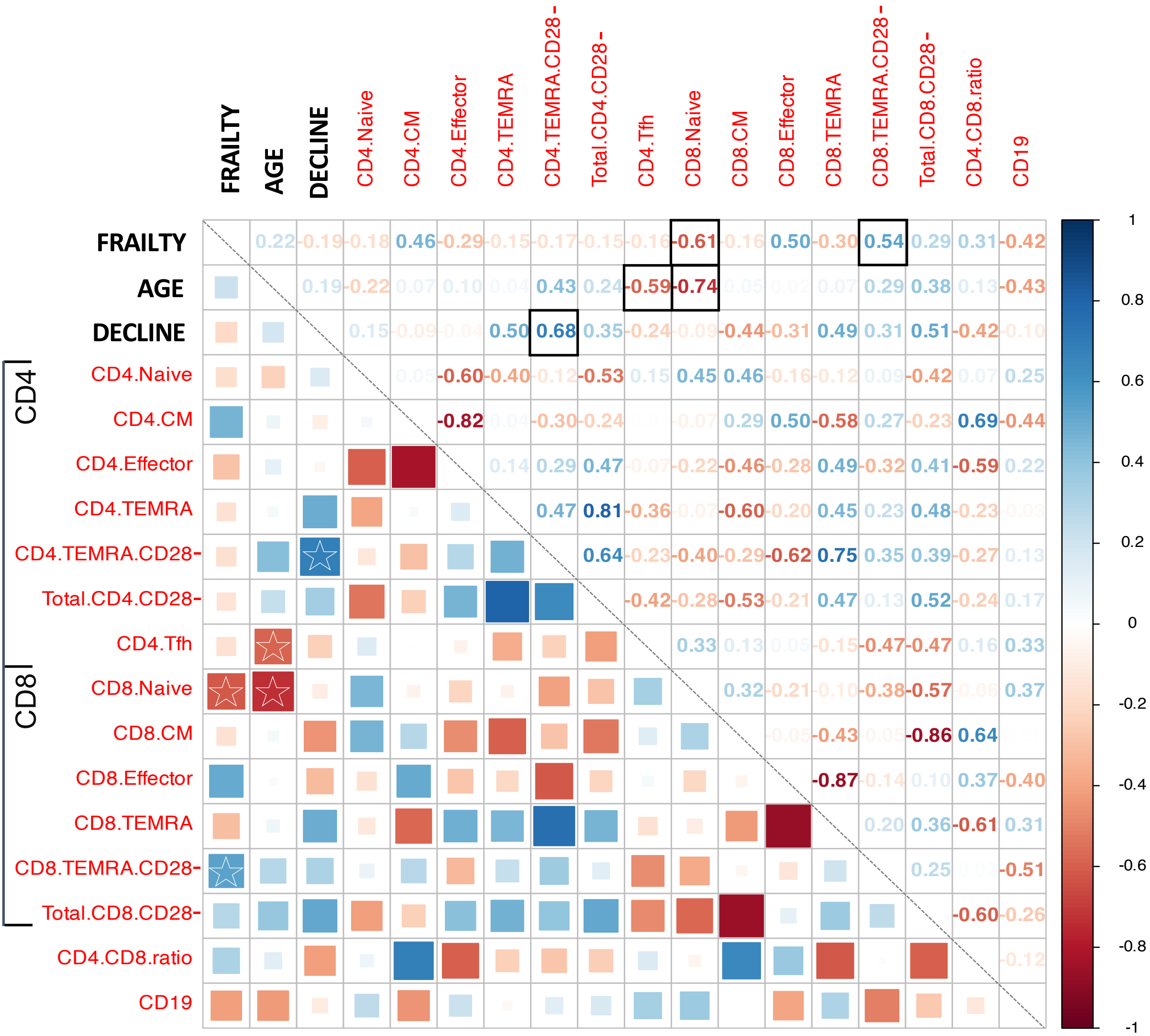
FRAILTY
AGE
DECLINE
CD4.Naive
CD4.CM
CD4.Effector
CD4.TEMRA
CD4.TEMRA.CD28-
Total.CD4.CD28-
CD4.Tfh
CD8.Naive
CD8.CM
CD8.Effector
CD8.TEMRA
CD8.TEMRA.CD28-
Total.CD8.CD28-
CD4.CD8.ratio
CD19
CD4
CD8
0.22 -0.19 -0.18 0.46 -0.29 -0.15 -0.17 -0.15 -0.16 -0.61 -0.16 0.50 -0.30 0.54 0.29 0.31 -0.42
0.19 -0.22 0.07 0.10 0.04 0.43 0.24 -0.59 -0.74 0.05 0.02 0.07 0.29 0.38 0.13 -0.43
0.15 -0.09 -0.04 0.50 0.68 0.35 -0.24 -0.09 -0.44 -0.31 0.49 0.31 0.51 -0.42 -0.10
0.05 -0.60 -0.40 -0.12 -0.53 0.15 0.45 0.46 -0.16 -0.12 0.09 -0.42 0.07 0.25
-0.82 0.04 -0.30 -0.24 -0.07 0.29 0.50 -0.58 0.27 -0.23 0.69 -0.44
0.14 0.29 0.47 -0.07 -0.22 -0.46 -0.28 0.49 -0.32 0.41 -0.59 0.22
0.47 0.81 -0.36 -0.07 -0.60 -0.20 0.45 0.23 0.48 -0.23 -0.03
0.64 -0.23 -0.40 -0.29 -0.62 0.75 0.35 0.39 -0.27 0.13
-0.42 -0.28 -0.53 -0.21 0.47 0.13 0.52 -0.24 0.17
0.33 0.13 0.05 -0.15 -0.47 -0.47 0.16 0.33
0.32 -0.21 -0.10 -0.38 -0.57 -0.06 0.37
-0.05 -0.43 -0.05 -0.86 0.64
-0.87 -0.14 0.10 0.37 -0.40
0.20 0.36 -0.61 0.31
0.25 -0.51
-0.60 -0.26
-0.12
1
0.8
0.6
0.4
0.2
0
-0.2
-0.4
-0.6
-0.8
-1

**Flow Cytometer Antibody Panel**		
**Marker**	**Fluor**	**Clone**
Live/Dead Zombie Aqua	BV510	
CD3	AF700	SK7
CD4	BV605	SK3
CD8	BV650	SK1
CD19	PerCP/Cy5.5	HIB19
CCR7	PE/Cy7	G043H7
CD45RA	ACP/Cy7	HI100
CXCR5	AF647	J252D4
CD28	BV711	CD28.2
CD69	KB520	FN50
PD1	PE	A17188B
CD137	PE/Dazzle 594	4B4-1
OX40	BV421	ACT35
